# Professor Jeeyon Jeong (1977-2024), an insightful scholar and generous mentor

**DOI:** 10.3389/fpls.2026.1814591

**Published:** 2026-05-08

**Authors:** Rachel A. Hendrickson

**Affiliations:** Amherst College Department of Biology, Amherst, MA, United States

**Keywords:** Arabidopsis, chromatin, ferroportin, homeostasis, iron

## Abstract

**Figure:**
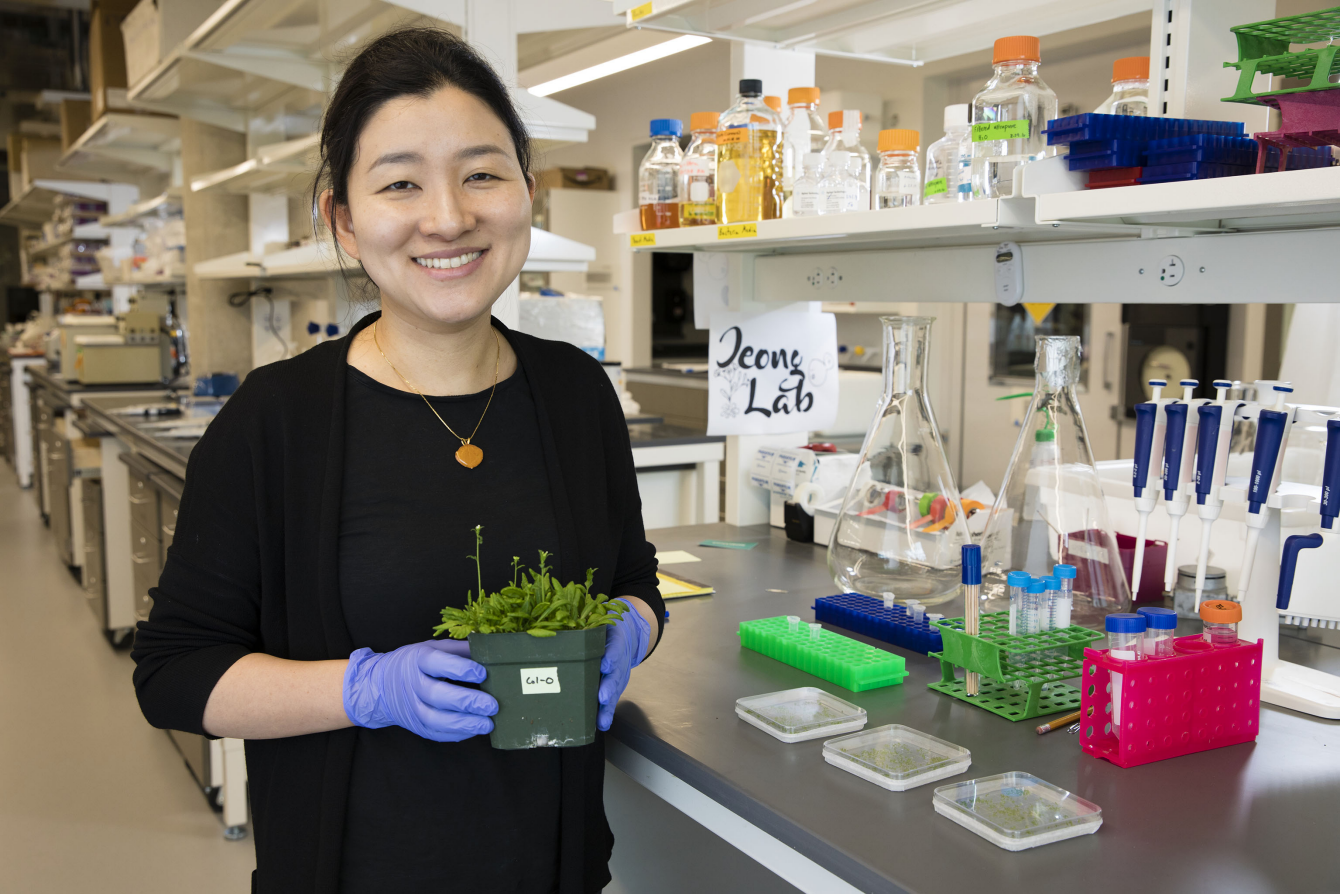
Professor Jeeyon Jeong in her lab at Amherst College. Photo credit: Maria Stenzel/Amherst College.

Professor Jeeyon Jeong in her lab at Amherst College. Photo credit: Maria Stenzel/Amherst College.

The Jeong Lab at Amherst College, established in 2015, has spent the past decade studying iron homeostasis in plants. Tragically, Professor Jeong passed away on October 14th, 2024. She is warmly remembered by her family, friends, colleagues, and students. I write this article as a member of the last cohort of students trained by Professor Jeong. In this article, I hope to review the scientific accomplishments of the lab and commemorate her legacy as a mentor and educator.

Born and raised in South Korea, Professor Jeong received her B.E. from Yonsei University and her M.S. from Pohang University of Science and Technology. She then emigrated to the United States for her PhD, during which she worked with Mary Lou Guerinot at Dartmouth to study iron homeostasis in plants. Until Jeeyon’s Ph.D. work, we did not know how chloroplasts take up iron, despite over 90% of the iron in plant leaves being found in the chloroplast. Her work provided strong evidence that iron needs to be reduced by a ferric chelate reductase encoded by *FRO7* before transport into the chloroplast (Jeong et al., 2008).

Professor Jeong then conducted her postdoctoral work at the University of Madison-Wisconsin studying zinc homeostasis in mammalian and yeast cells with David Eide. As a postdoc, Jeeyon’s work overturned the prevailing theory as to how mutations in the zinc transporter encoded by *ZIP13* cause the spondylocheiro dysplastic form of Ehlers–Danlos syndrome (SCD-EDS), a heritable connective tissue disorder. Jeeyon showed ZIP13 functions to release labile zinc from vesicular stores for use in the ER and other compartments and proposed that SCD-EDS is the result of vesicular zinc trapping and ER zinc deficiency rather than ER zinc overload as had been suggested earlier (Jeong et al., 2012).

Professor Jeong (**Graphical Abstract**) joined the faculty of Amherst College in 2015 as a member of the Biology department and Biochemistry & Biophysics program. At Amherst, Professor Jeong taught Molecular Genetics, Biochemistry, an introductory molecular biology course, an advanced seminar on metals in biology, and, most recently, a course focused on biology outreach through community learning.

In starting her lab, Professor Jeong returned to her roots researching the regulation of iron in plants using molecular genetic and bioinformatic approaches. To put Professor Jeong’s lab projects in context, more than 2 billion people are iron deficient because their plant-based diets are not a rich source of this micronutrient. Clearly, we need to understand iron homeostasis in plants, from the point of view of both improving plant growth and crop yields as well as improving human nutrition. Iron is an essential micronutrient in plants, serving as a key redox cofactor in photosynthesis and cellular respiration. However, when present in excess, iron can induce toxicity through oxidative stress. While iron is an abundant element in soil, it is primarily found in its insoluble, ferric form (Colombo et al., 2014; Li et al., 2024). To combat the resulting limited bioavailability, *Arabidopsis thaliana* uses the reduction-based iron acquisition method Strategy I, which involves rhizosphere acidification, reduction of ferric iron, and subsequent import of ferrous iron (Jeong et al., 2017). Once in the roots, iron is transported to the vasculature and the shoots and may be directed to developing organs and seeds. Proper iron localization, critical for chloroplast and mitochondria function, is mediated by transporters. Within Arabidopsis, intricate transcriptional regulatory networks ensure appropriate iron acquisition and regulation in response to external iron availability (Riaz and Guerinot, 2021). Recently, Professor Jeong proposed that epigenetic mechanisms regulating iron homeostasis represent the next frontier in understanding plant iron homeostasis (Su et al., 2022).

As Professor Jeong started her lab at Amherst, she and her students primarily focused on determining the function of the protein Ferroportin3. Ferroportin (FPN) proteins are iron transport proteins found in vertebrates, plants, and at least one bacterial species (Drakesmith et al., 2015; Taniguchi et al., 2015). In Arabidopsis, three FPNs have been identified: FPN1, which transports iron from the root vasculature into the xylem, FPN2, which imports iron into the vacuole in the plant cortex, and FPN3 (Morrissey et al., 2009). FPN3 was initially characterized in 2009 as MULTIPLE ANTIBIOTIC RESISTANCE 1 (MAR1), a chloroplast-localized protein whose presence sensitized plants to antibiotics (Conte et al., 2009; Conte and Lloyd, 2010). Predicted transit peptides from the ARAMEMNON suggest mitochondrial or chloroplast localization (Schwacke et al., 2003), and confocal microscopy conducted by Jeong Lab students showed colocalization of GFP-tagged FPN3 with plastids and mitochondria (Kim et al., 2021). To assess the direction of iron transport, FPN3 was introduced into yeast carrying mutations in various mitochondrial iron importers and compared to yeast overexpressing the yeast mitochondrial iron exporter (Kim et al., 2021). Yeast with FPN3 introduced to mitochondria had similar phenotypes to addition of yeast mitochondrial iron exporters MMT1/2, and exacerbated phenotypes where mitochondrial iron importer-encoding genes *MRS3/4* were knocked out (Kim et al., 2021). Through these experiments, the directionality of FPN3 as a mitochondrial and chloroplast iron-exporter was elucidated. This was confirmed through analysis of iron content in isolated chloroplasts and mitochondria from *fpn3* knockout plants, which was significantly higher than in Wild-type (Kim et al., 2021).

With FPN3’s role as a dual-targeted mitochondrial and chloroplast iron exporter specified, research in the Jeong Lab has focused more recently on the physiological significance of FPN3. Through the lab’s work on FPN3, two major threads have arisen: the importance of FPN3 in iron deficient conditions and the functional interaction of FPN3 with other proteins involved in subcellular iron regulation.

The importance of FPN3 during iron deficiency became clear as Jeong Lab members assessed the tissue-scale localization of FPN3. *FPN3* is expressed in shoots, flowers, siliques, and the root cap regardless of iron conditions. In roots, however, the lab found that *FPN3* expression only occurred in iron-deficient conditions, with expression regulated via a local root signal (Kim et al., 2021). Overall growth of *fpn3* seedlings was shown to be decreased under iron deficiency, and transmission electron microscopy (TEM) also revealed altered mitochondrial morphology in *fpn3* plants under iron deficiency (Kim et al., 2021). Together, these results suggested that FPN3 is particularly important under iron deficiency, and the lab continued to investigate potential mechanistic underpinnings for the observed phenotypes. Recent work has been particularly focused on another phenotype found during TEM of *fpn3* roots under iron deficiency: enlarged plasma membrane invaginations (unpublished data). Iron-regulated transporter 1 (IRT1) is a high-affinity iron transporter responsible for iron import into the root from the rhizosphere (Eide et al., 1996). IRT1 is post-translationally regulated by removal from the plasma membrane via ubiquitination and endocytosis (Ivanov et al., 2014), especially in the presence of excess non-iron metals (Dubeaux et al., 2018). Given the TEM findings, it was hypothesized that FPN3 may be involved in maintaining proper IRT1 cycling. Preliminary observations show that without FPN3, plants no longer exhibit the changes in IRT1 localization in response to different iron and metal conditions seen in Wild-type seedlings. Another recent hypothesis regarding the origin of the observed plasma membrane invaginations was that they may be caused by dysregulation of suberin deposits in the *fpn3* knockout plants under iron deficient conditions.

As FPN3 facilitates subcellular iron localization, the Jeong Lab researched higher-order mutant lines in which both *FPN3* and genes involved in the mitigation of cellular iron toxicity were knocked out. In Arabidopsis, the vacuole plays a significant role in iron sequestration (Bashir et al., 2016). Iron is imported into the vacuole by vacuolar iron transporter 1 (VIT1) (Kim et al., 2006). To investigate if FPN3’s importance may be exacerbated without available iron stores in the chloroplast, mitochondria, and vacuole, an *fpn3 vit1* line was developed, with its behavior under iron deficiency of particular interest. The lab found that in alkaline soil, the growth and shoot weight of *fpn3 vit1* plants surpassed that of *fpn3* and *vit1* single mutant plants (unpublished data). The improvement was especially fascinating given VIT1 has been shown to be necessary for growth on alkaline soil, with marked decrease in growth and extreme chlorosis (Kim et al., 2006). A major avenue to try and understand this phenotype was through assaying Strategy I iron acquisition in the double mutant, as increased acquisition could potentially account for this recovery. Activity of the protein responsible for rhizosphere acidification, Arabidopsis plasma membrane H+-ATPase2 (AHA2), was one aspect of Strategy I the lab investigated in *fpn3 vit1* (Santi and Schmidt, 2009). Students initially used an in-gel assay to assess rhizosphere acidification with the pH indicator bromocresol purple, however qualitative analysis of individual seedlings proved variable. To address this, students in the lab developed a quantified rhizosphere acidification assay to analyze bulk samples in a way that would allow for statistical comparisons (Omer et al., 2023). In addition to *fpn3 vit1*, the lab has recently been focused on research of plants with both *FPN3* and iron-binding ferritin (*FER*) genes knocked out. As ferritins have been shown to bind mitochondria and plastid iron to prevent oxidative stress, loss of *FPN3* and *FER1*, *FER3*, and *FER4* would hypothetically increase the labile iron in both chloroplast and mitochondria and provide insight into the functional interaction of FPN3 and the ferritins (Ravet et al., 2009; Briat et al., 2010). Interestingly, a cotyledon-specific variegation phenotype was observed in 39% of seedlings in *fpn3 fer1-3–4* iron sufficient conditions and 34% of seedlings in iron excess conditions (unpublished data). Phenotypic and transcriptomic studies of *fpn3 fer1-3–4* seedlings have been conducted, as well as experiments assaying oxidative stress and chloroplast function in these plants.

In addition to the study of FPN3, Professor Jeong was also involved in the study of epigenetic regulation of the iron deficiency response in Arabidopsis in a collaborative project with her husband, Joohyun Lee. Polycomb Repressive Complex 2 (PRC2)-mediated trimethylation of Histone 3 lysine 27 (H3K27me3) acts as a repressive mark in *Arabidopsis thaliana* by inducing transcription-preventing chromatin compaction (Köhler and Hennig, 2010; Pu and Sung, 2015; Margueron and Reinberg, 2011). Work by Jeong Lab students helped to identify H3K27me3 as involved in the repression of the iron deficiency response. To study H3K27me3, students in the lab used seedlings without the CURLY LEAF (CLF) subunit of PRC2 (Chanvivattana et al., 2004; Schubert et al., 2006; Zhang et al., 2007), a mutation that has been shown to significantly inhibit methyltransferase activity and has been used as a PRC2 mutant (Lafos et al., 2011; Lu et al., 2011; Zhou et al., 2018). Under iron deficiency, the basic Helix-Loop-Helix (bHLH) transcription factor FER-LIKE IRON DEFICIENCY-INDUCED TRANSCRIPTION (FIT) dimerizes with other bHLH transcription factors to induce transcription of genes involved in iron acquisition as well as itself, creating a positive feedback loop (Colangelo and Guerinot, 2004; Jakoby et al., 2004). The lab found H3K27me3 deposition on FIT and FIT-dependent genes and increased expression of these genes in *clf* mutant plants (Park et al., 2019). These findings were specific to the FIT-dependent iron deficiency response, as genes involved in other aspects of the iron deficiency response – including POPEYE (PYE) and BRUTUS (BTS) (Long et al., 2010; Hindt et al., 2017; Rodríguez-Celma et al., 2019) – were not found to be impacted by H3K27me3 (Park et al., 2019). Continued work by Jeong Lab students also discovered H3K27me3 to be involved in shoot iron homeostasis, with differential iron translocation patterns found in *clf* mutant plants relative to Wild-type seedlings (Park et al., 2020). Further, genes involved in shoot-root iron signaling, *YELLOW STRIPE LIKE 1* (*YSL1*) and *IRONMAN 1* (*IMA1*) were found to be regulated by H3K27me3 (Waters et al., 2006; Grillet et al., 2018; Park et al., 2020). To aid in the continued characterization of how H3K27me3 interacts with the FIT-dependent iron deficiency response, *FIT/bHLH39* overexpressing lines were developed and investigated in both a WT and *clf* mutant background (unpublished data). These investigations into H3K27me3 have developed and complicated our understanding of iron deficiency regulation.

The contributions of the Jeong Lab have been immensely valuable scientifically, expanding our knowledge of and appreciation for the intricate molecular mechanisms governing how Arabidopsis regulates iron. Just as significant as the lab’s scientific progress is how it came to be: the training by Professor Jeong of almost fifty undergraduates and research technicians with attention, excitement, and dedication. Many students in the lab enter with no experience in biological research. Thus, while students contributed to a growing understanding of iron homeostasis, they were also learning what it means to be a scientist.

Professor Jeong cared deeply for her science and for her students; I could not be more grateful to have spent my undergraduate years learning from her. When I entered the lab, I began a microscopy project distinct from what other students in the lab were focused on; Professor Jeong trusted me, as she trusted many students in the lab, to read papers, design and execute experiments, and ask interesting questions. Her high expectations were what enabled me to meet them. I was given room to grow and room to fail. No matter the time of day, 2 pm or 2 am, Professor Jeong was there to answer an emailed question or provide a round of edits on a draft sent just thirty minutes earlier. During weekly lab meetings, each student took on a figure from a paper to present. Through this, students were quickly introduced to the ins and outs of the field, common molecular biology techniques, and the new results that might shape how we approach our own research. Other meetings involved preparing students for presentations of their research; all students were encouraged to ask questions that might help prepare for the harshness of a committee meeting. Most students in the lab worked on different projects, taking on different angles or approaches to tackle one of the overarching questions on the roles of FPN3 and H3K27me3. Professor Jeong was always ready to push students one step further: to encourage them to come to a conference, apply to an REU or fellowship, conduct a special topics research course or thesis, contribute to the writing of a mini-review or paper, or present their research to a collaborator or at a conference. I cannot count the number of things I never would have dreamed of accomplishing if not for Professor Jeong’s encouragement and support.

As Amherst College is a primarily undergraduate institution, most research in the Jeong Lab is conducted by students who join the lab for relatively short periods of time. The lab’s research has thus evolved through being touched by many hands and filtered through many minds. For example, research into a single protein, FPN3, grew in many different directions as students tested various hypotheses. Lab members studied seemingly distinct questions regarding FPN3’s physiological significance alongside one another, contextualizing their own work within the greater work of the lab and the field. Students gained an appreciation for how FPN3’s various connections to iron homeostatic pathways all fit together. Truly comprehending how a single protein could be involved in so many processes on molecular, cellular, and organismal scales has meant opening my eyes to the beauty of biology.

Professor Jeong prepared us to nurture the next generation of scientists. In addition to the articles, reviews, and a protocol published in plant biology journals, students in the lab authored many publications in journals designed for kids and teens, highlighting both the work done by the lab to advance our understanding of iron in plants as well as why that work is significant and interesting. Students in the lab also participated in teaching science at the local elementary school, visits which were recently formalized as Professor Jeong began a course in the biology department focused on community engagement. In the lab, Professor Jeong strongly encouraged students who had been working for a year to begin training and support students with less experience.

As I leave Amherst, it is easy to become caught up on questions of legacy, of how this work will continue to live on. Of course, there is Amherst itself, touched by Professor Jeong’s presence in ways I am certain will last for many years. Then there is her science, which will continue to be advanced through the work of other dedicated researchers. And finally, there are the many students trained in the lab with rich lives of inquiry and exploration ahead of them.

## In memoriam

Dedicated to Professor Jeeyon Jeong (1977-2024), an insightful scholar and generous mentor.
